# Relaxivity of Gadobutrol and Gadoteric Acid in Cerebrospinal Fluid at 3T

**DOI:** 10.1002/mrm.70284

**Published:** 2026-02-01

**Authors:** Sofia Behndig, Anders Garpebring, Daniel Dahlgren Lindström, Jan Malm, Anders Wåhlin, Anders Eklund

**Affiliations:** ^1^ Department of Diagnostics and Intervention Biomedical Engineering and Radiation Physics, Umeå University Umeå Sweden; ^2^ Department of Diagnostics and Intervention, Diagnostic Radiology Umeå University Umeå Sweden; ^3^ Department of Clinical Sciences, Neurosciences Umeå University Umeå Sweden; ^4^ Umeå Center for Functional Brain Imaging (UFBI), Umeå University Umeå Sweden; ^5^ Department of Applied Physics and Electronics Umeå University Umeå Sweden

**Keywords:** cerebrospinal fluid, MRI contrast media, phantom, relaxivity, T1 mapping

## Abstract

**Purpose:**

The aim was to estimate T1 relaxivity of gadobutrol and gadoteric acid in cerebrospinal fluid (CSF) at 3T, to support research on CSF‐flow and the glymphatic system in humans utilizing T1 mapping after intrathecal injection.

**Methods:**

Using a phantom, relaxivity was estimated for gadobutrol and gadoteric acid in lumbar CSF and an isotonic solution. All samples were scanned simultaneously using the variable flip angle method with B1 correction, repeated six times on one 3T scanner, and once on a second 3T scanner. Difference in relaxivity between CSF and the isotonic solution were evaluated from the repeated measurements.

**Results:**

There was a significant difference in relaxivity between CSF and the isotonic solution for both gadobutrol and gadoteric acid. The relaxivity for gadobutrol for the respective scanners was estimated to 3.02 ± 0.09 vs. 3.63 L mmol^−1^ s^−1^ in CSF and 2.35 ± 0.05 vs. 2.74 L mmol^−1^ s^−1^ in isotonic solution. For gadoteric acid, corresponding results were 2.47 ± 0.02 vs. 2.91 L mmol^−1^ s^−1^ in CSF and 2.37 ± 0.03 vs. 2.8 L mmol^−1^ s^−1^ in isotonic solution. Between the scanners, there was a high correlation (*R* ^2^ 0.998) but an 18% scaling difference in the T1 relaxation rates and corresponding relaxivities.

**Conclusions:**

The relaxivity was higher in CSF than in the isotonic solution, particularly for gadobutrol. Systematic differences in relaxivity between scanners may potentially be corrected using a scaling factor derived from the T1 time of baseline CSF. For CSF studies using T1 mapping with a gadolinium‐based contrast agent, we recommend using a CSF‐specific relaxivity constant.

## 1 Introduction

In MRI, a gadolinium‐based contrast agent (GBCA) can be used to give a change in image contrast, with the size of this change being dependent on the gadolinium (Gd)‐concentration and the GBCAs relaxivity. In clinical trials, MRI has been applied before and after off‐label intrathecal injection of a GBCA to study the flow of cerebrospinal fluid (CSF) in humans [1, 2, 3, 4]. Research about CSF‐flow and distribution has gained interest with the so called glymphatic system, a proposed brain waste clearance system [5], hypothesized to be relevant to the pathophysiology of several neurological diseases, including Alzheimer's disease [6]. Several aspects of the glymphatic system are still unknown, including driving mechanisms and flow pathways. By applying T1 mapping before and at multiple timepoints after intrathecal injection of a GBCA, the gadolinium (Gd)‐concentration can be quantified and compared between brain tissue and CSF [1], enabling modeling of the glymphatic flow. The Gd‐concentration can be calculated from equation (1):

(1) $$\frac{1}{T_{1}\left(t\right)}-\frac{1}{T_{10}}=C\cdot r_{1}$$

where *T*_1_(*t*) is the T1 time at a post‐injection time point, *T*_10_ is the baseline (pre‐injection) T1 time, *C* the Gd‐concentration, and *r*_1_ the T1 relaxivity, a parameter describing the magnetic properties of the GBCA. Correct calculation of the Gd‐concentration is thus dependent on the relaxivity, which in turn is dependent on several factors, including temperature, magnetic field strength, and medium it is measured in. The relaxivity has previously been estimated for whole blood, plasma, and water [7, 8, 9, 10, 11], but not for CSF.

The aim of this study was to estimate *r*_1_ of two GBCAs in CSF at 3T. For comparability with previous studies, *r*_1_ was also estimated in an isotonic solution expected to have similar properties to CSF. As a secondary objective, we compared the intra‐scanner reproducibility, as well as the results between two different scanners.

## 2 Methods

We estimated *r*_1_ of the two GBCAs gadobutrol (GADOVIST, Bayer Pharma, Berlin, Germany) and gadoteric acid (DOTAREM, Guerbet, Rossy CdG Cedex, France) in CSF and an isotonic solution (RINGER‐ACETAT BAXTER VIAFLO, Baxter, Kista, Sweden) using a phantom. For comparability, both GBCAs in both mediums were scanned simultaneously. Measurements were done on two different 3T scanners using the variable flip angle (VFA) method [12] with B1 correction. The data set and scripts used in the study can be found on Github (https://github.com/SofiaBehndig/CSF_relaxivity).

### 2.1 Cerebrospinal Fluid

The study was approved by the Swedish Ethical Review Authority. All participants gave written and oral informed consent before inclusion. CSF was acquired from patients who were scheduled for an external lumbar drainage as part of their investigation for suspected idiopathic normal pressure hydrocephalus (iNPH). Exclusion criteria were age > 65. During external lumbar drainage, CSF is continually drained to a closed system over a period of 3 days, after which it was stored in a −80°C freezer. Before storage, the CSF was analyzed for albumin, erythrocytes, and cells, and discarded if the result were outside the reference interval of our laboratory: albumin level < 360 mg/L, erythrocyte count < 300 cells/μL, mononuclear cell count < 5 cells/μL, polynuclear cell count < 1 cell/μL. The CSF used in the study had an albumin level of 244.5 ± 94.05 mg/L, erythrocyte count of < 300 cells/μL, mononuclear cell count of 1 ± 1.41 cells/μL, and polynuclear cell count of 0 cell/μL.

On the day of the MRI, the CSF was taken out of the freezer and thawed in room temperature. Equal portions of CSF from two different subjects (26 mL each) were mixed before they were diluted with either gadobutrol or gadoteric acid. Samples were diluted to contain Gd‐concentrations of 0, 145, 289, 427, 572, 709, 854, and 999 μM. Prior to dilution, the standard deviation of the pipets had been investigated. Based on this investigation, we calculated absolute errors of the Gd‐concentration according to the Gaussian law of error propagation, giving errors of less than ±0.3%. The concentrations were chosen to be in the range of estimated Gd‐concentrations in CSF as found after intrathecal injection [1].

### 2.2 Isotonic Solution

The isotonic solution was diluted with gadobutrol and gadoteric acid separately as described above for CSF. The specific isotonic solution was chosen as it has a similar ion composition as that of CSF [13, 14].

### 2.3 Phantom

**FIGURE 1:**
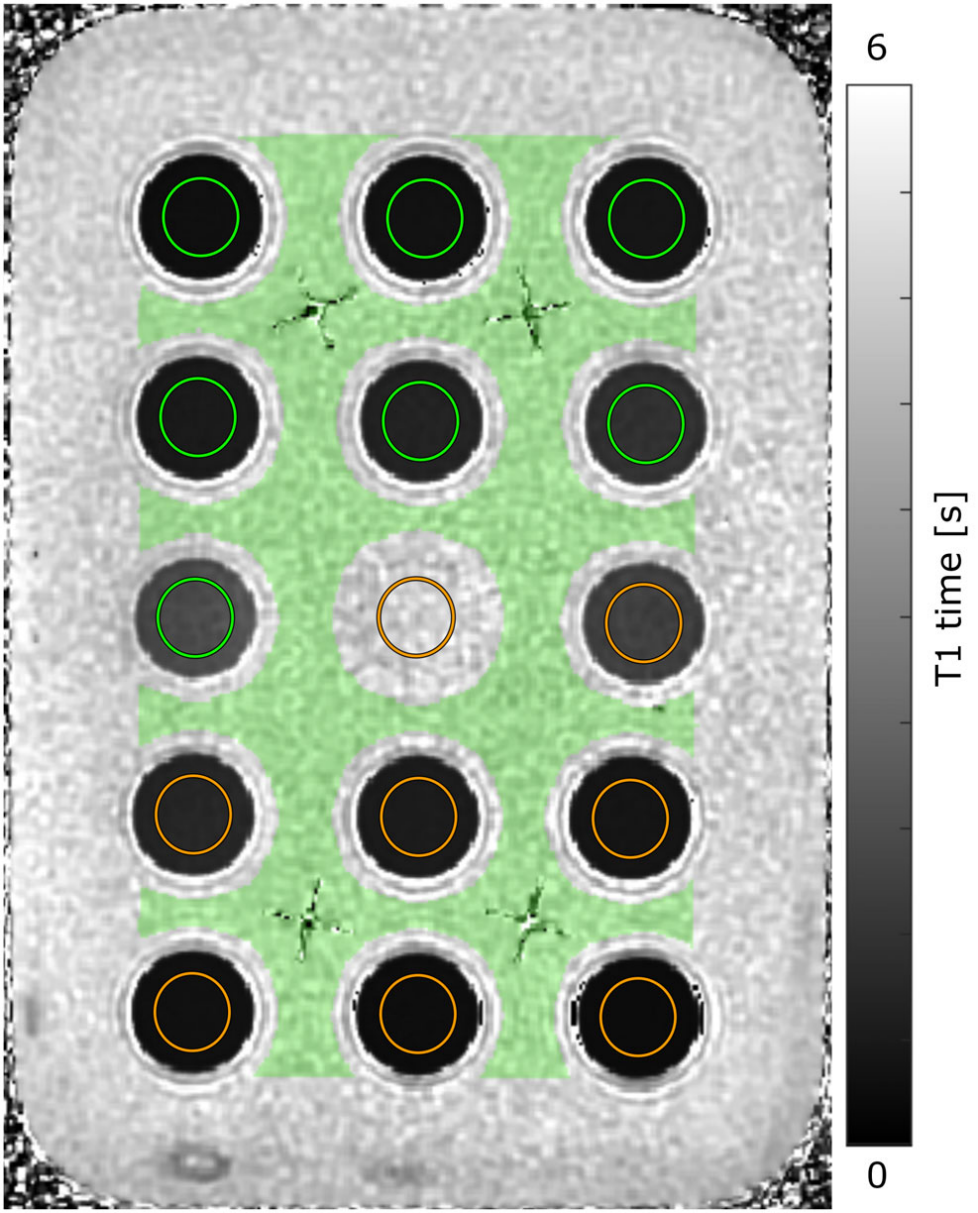
T1 map of the phantom. The same phantom contained samples of cerebrospinal fluid (orange) or an isotonic solution (green), diluted with gadobutrol, or gadoteric acid. The phantom was divided into two layers, one contrast agent in each. Each layer contained 16 samples, eight for each medium, diluted to gadolinium concentrations between 0–1 mM. The fluid surrounding the samples made out the 0 mM sample for the isotonic solution. The colored circles and area illustrate the regions of interest used for analysis. Note that the center orange circle contains the isolated CSF sample with 0 mM of Gd, however not visibly different from the isotonic solution with 0 mM of Gd on the T1 map.

The phantom was constructed from 3D‐printed vials and a plastic container filled with the isotonic solution (Figure 1). The vials had an inner diameter of 18 mm and a height of 13 mm. Each vial was filled with one dilution of either CSF or isotonic solution. The vials were positioned in two levels, one with gadobutrol and one with gadoteric acid, with 15 samples per level. All vials were then put in the plastic container filled with the isotonic solution, which made up the undiluted sample containing 0 μM of Gd. The 0 μM of Gd‐samples will henceforth be referred to as baseline isotonic solution or CSF, respectively.

To account for T1 variability with temperature, the phantom was kept in Styrofoam and heated to approximately 37.5°C before each scan. To evaluate for temperature variation, an MRI compatible fiber optic thermometer (Luxtron M604, LumaSense technologies, Santa Clara, California) was put in the phantom to measure the temperature during the scans. The mean temperature of all scans was 36.9°C ± 0.47°C with an average temperature drop of 0.037°C ± 0.007°C/min. Change in temperature with respect to time was derived from the slope of the linear regression between temperature and time.

### 2.4 Imaging Parameters

The phantom was scanned in two different 3T scanners. Scanner A (Signa; GE Healthcare, Milwaukee, Wisconsin) had a 16‐channel head coil, software versions MP26.0_R03_2125.b, and Scanner B (Signa Premier; GE Healthcare, Milwaukee, Wisconsin) had a 48‐channel head coil, software versions 30∖LX∖SIGNA_LX1.MR30.1_R01_2322.c. T1 mapping was performed using the VFA method [12]. VFA images were acquired with a 3D fast spoiled gradient echo sequence using five different flip angles with isotropic acquisition of 1 mm, 100 slices, field of view 256 × 256, TR = 7.2, TE = 2.9, with flip angles acquired in the following order *α*_1_ = 12°, *α*_2_ = 2°, *α*_3_ = 7°, *α*_4_ = 16°, *α*_5_ = 20°. For Scanner A, TR could not be set to a fixed value, and instead ranged between 7.18–7.28 ms depending on the flip angle. As the VFA method is sensitive to B1‐inhomogenisities, B1 mapping was further performed using the Bloch‐Siegert shift approach [15] as implemented by the vendor, with *α* = 20, slice thickness 3 mm, and in‐plane resolution of 4 mm. Total scan time was approximately 17 min. To evaluate the reproducibility of the VFA method and the corresponding *r*_1_ estimation, the imaging sequence was repeated six times using Scanner A, with a new localizer performed before each separate repetition. To limit potential effects caused by degeneration of the GBCAs after breakage of the vials' seals [16], all scans on both Scanner A and B were completed within 12 h to the beginning of the dilution process of the CSF and isotonic solution samples.

### 2.5 B1 Map

Prior to flip angle correction, the B1 map was processed in two separate steps. First, to eliminate noisy data due to signal distortion caused by the phantom's plastic container and support structures (Figure 1), outlier voxels within the phantom were replaced through interpolation, followed by median filtering. Using the noise‐eliminated B1 map, we still observed a gradient in derived T1 times of the baseline isotonic solution within and across slices. Therefore, based on the assumption that the T1 time should be uniform in the baseline isotonic solution, a second correction step was introduced. To model the additional B1 correction required to adjust for the observed T1 trend across the phantom, voxel‐wise correction factors forcing T1 uniformity on the baseline isotonic solution were calculated, and then fitted to a second‐degree polynomial. From this polynomial, a smoothed correction map could be generated for the entire phantom before being added to the noise‐eliminated B1 map, producing a trend‐corrected B1 map that better accounted for inhomogeneities in the B1 field. The forced T1 time was determined as the mean of the baseline isotonic solution as found after the first correction step.

### 2.6 T1 Map

T1 maps were generated per voxel from the five flip angle images using a nonlinear least squares method optimized using the Levenberg–Marquardt method. The flip angle specific TRs were used in the optimization. With the total scan time for acquiring five flip angles reaching 17 min, repeated measures as needed to study CSF‐flow in humans [1, 17] become less feasible. To simulate a more clinically useful setting, a separate T1 map using only two flip angle images (*α*_1_ and *α*_2_) was further generated.

For every T1 map, a separate region of interest (ROI) was generated for each sample (Figure 1). For the samples in the 3D‐printed vials (all samples except the baseline isotonic solution), a spherical ROI was created and then eroded to eliminate voxels possibly affected by the border of the vial. The ROI for the baseline isotonic solution was generated using a similar approach, eliminating any border effects both from the container and the vials. Even so, there were a few voxels remaining in the baseline isotonic solution ROI with unreasonable T1 times, why voxels outside 0 to 10 000 ms were excluded from analysis, removing about 0.2% of the voxels from the original ROI. All ROIs were three slices thick.

### 2.7 Statistical Analysis

The relaxivity was calculated from the slope of the linear regression between the relaxation rate (1/T1) and the concentration of the GBCA. Differences in relaxivity of gadobutrol and gadoteric acid in CSF to the isotonic solution were tested for the repeated measures using an independent sample *t*‐test. Differences between estimated relaxivities from the T1 map generated from five, respectively, two flip angles were tested using a paired sample *t*‐test. The relationship between the two scanners was tested using linear regression. Possible differences between the scanners were analyzed. Results are presented as means and standard deviations unless stated otherwise. All statistical tests were performed in IBM SPSS Statistics (IBM SPSS Statistics for Windows, Version 27.0. Armonk, NY: IBM Corp). A *p*‐value of < 0.05 was considered statistically significant.

## 3 Results

### 3.1 Relaxivity

**FIGURE 2:**
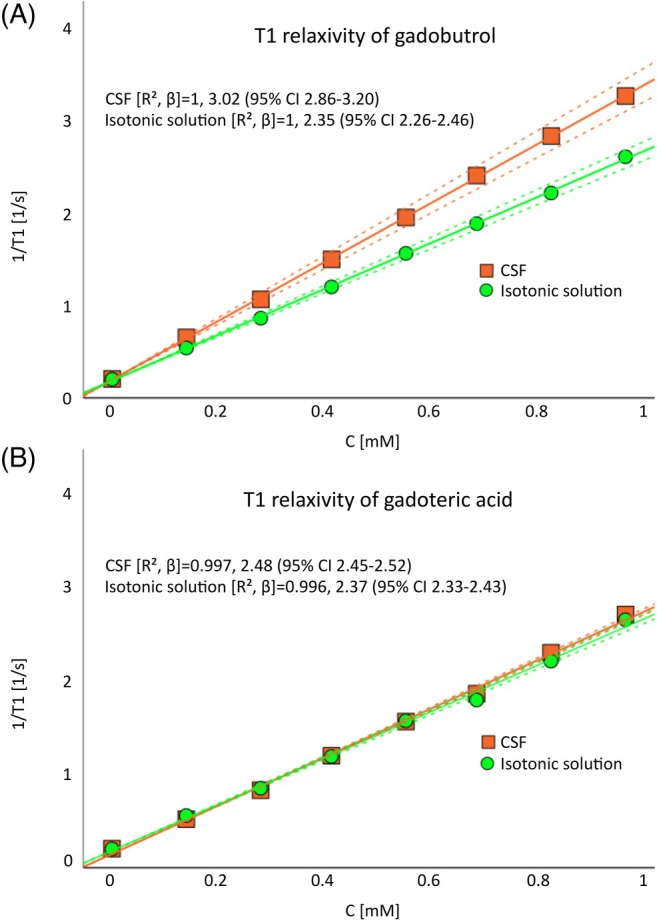
Estimations of T1 relaxivity for gadobutrol and gadoteric acid in cerebrospinal fluid (CSF) and an isotonic solution. The figure represents the results from Scanner A reconstructed from all five flip angles. The relaxivity was calculated from the slope of the linear regression between calculated T1 relaxation rates (1/T1) versus the gadolinium concentration. The dotted lines illustrated the 95% confidence interval (CI) of the repeated measures. There was a difference in relaxivity between CSF and the isotonic solution both for gadobutrol (*p* < 0.001, mean difference 0.67, 95% CI 0.58–0.76) and gadoteric acid (*p* < 0.001, mean difference 0.1, 95% CI 0.08–0.13). (C, gadolinium concentration; *R* ^2^, coefficient of determination; *β*, slope).

The relaxivity of the two GBCAs in CSF and the isotonic solution estimated from Scanner A are presented in Table 1 and Figure 2. There was a significant difference in *r*_1_ between CSF and the isotonic solution for both gadobutrol (*p* < 0.001, five flip angles mean difference 0.67, 95% confidence interval 0.58–0.76) and gadoteric acid (*p* < 0.001, five flip angles mean difference 0.1, 95% CI 0.08–0.13).

**TABLE 1:** Relaxivity of gadobutrol and gadoteric acid in CSF and an isotonic solution for Scanner A.

	Gadobutrol		Gadoteric acid	
	CSF	Isotonic solution	CSF	Isotonic solution
Five flip angles	3.02 ± 0.09	2.36 ± 0.05	2.48 ± 0.02	2.38 ± 0.03
Two flip angles	3.40 ± 0.08	2.44 ± 0.02	2.50 ± 0.02	2.55 ± 0.02
*p*‐value^a^	< 0.001	0.002	0.02	< 0.001

*Note*: The relaxivity is presented in the unit of L mmol^−1^ s^−1^.

Abbreviation: CSF, cerebrospinal fluid.

^a^ Paired *t*‐test between five and two flip angles are based on partially overlapping datasets, affecting independence and significance.

### 3.2 Scanner Comparison

**FIGURE 3:**
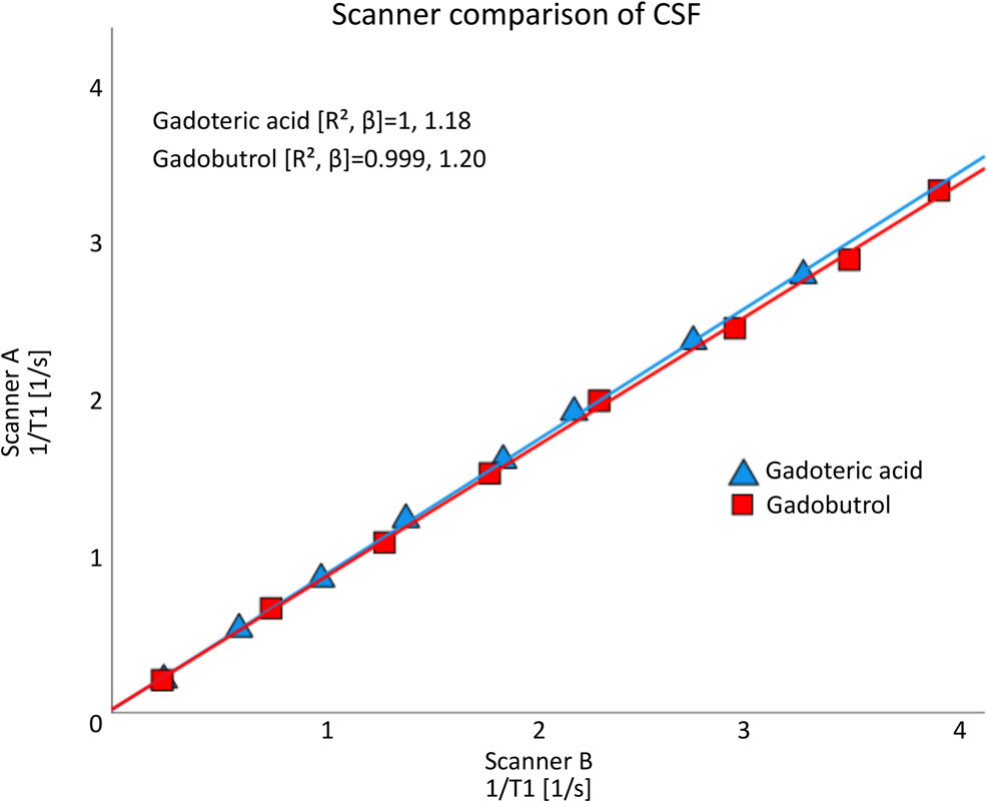
Comparison of T1 relaxation rates (1/T1) measured by Scanner A and B. The comparison was made using samples of cerebrospinal fluid (CSF) diluted with either gadoteric acid or gadobutrol, with gadolinium concentration ranging between 0–1 mM. There was a systematic difference between the two scanners, with an average difference in 1/T1 of 19% for the CSF samples. (*R* ^2^, coefficient of determination; *β*, slope).

The *r*_1_ estimates for Scanner B are presented in Table 2, and for the five flip angle image they were on average 18% higher than for Scanner A. This difference was also reflected in the T1 relaxation rate (1/T1), where values calculated for Scanner B (in both CSF and the isotonic solution) were on average 18% higher than those calculated from Scanner A (Figure 3, *R* ^2^ 0.998–1, β 1.17–1.20). The baseline T1 times differed on average by 17% between the scanners (Table 3).

**TABLE 2:** Relaxivity of gadobutrol and gadoteric acid in CSF and an isotonic solution for Scanner B.

	Gadobutrol		Gadoteric acid	
	CSF	Isotonic solution	CSF	Isotonic solution
Five flip angles	3.64	2.75	2.92	2.81
Two flip angles	3.65	2.84	2.91	2.76

*Note*: The relaxivity is in the unit of L mmol^−1^ s^−1^.

Abbreviation: CSF, cerebrospinal fluid.

**TABLE 3:** T1 times of baseline CSF and isotonic solution.

	Scanner A		Scanner B	
	CSF	Isotonic solution	CSF	Isotonic solution
Five flip angles [ms]	4957 ± 92	5028 ± 57	4254	4335
Two flip angles [ms]	4961 ± 105	5041 ± 42	4216	4290

Abbreviation: CSF, cerebrospinal fluid.

To assess how much of the difference in estimated *r*_1_ could be attributed to this systematic difference in measured T1 times, we applied a proportional scaling model according to equation (1). Assuming that the proportional difference in 1/T1 is concentration‐independent, we could calculate a scaling factor (κ) based on the T1 time of baseline CSF:

$$\kappa =\frac{1/T_{10}^{B}}{1/T_{10}^{A}}$$

Using this factor, the adjusted *r*_1_ of gadobutrol on Scanner B should be:

$$\begin{array}{c} r_{1}^{B}={\kappa r}_{1}^{A}=3.52\;L\;{\text{mmol}}^{-1}s^{-1} \end{array}$$

This is close to the estimated *r*_1_ on Scanner B (3.64 L mmol^−1^ s^−1^).

Similarly, for the two flip angle image, *r*_1_ for Scanner B was on average 13% higher than for Scanner A where the corresponding T1 relaxation rates were on average 12% higher (*R* ^2^ 0.998–1, β 1.07–1.16). The baseline T1 times differed on average by 18% between the scanners (Table 3).

## 4 Discussion

We present the T1 relaxivity (*r*_1_) in CSF and an isotonic solution in a physiological temperature for two GBCAs at 3T using the VFA method, with repeated measurements on one MR scanner (A). These repeated measures had a high reproducibility, which is consistent with previous studies [18, 19]. Our results show that *r*_1_ is higher in CSF compared to an isotonic solution, with the difference being more relevant for gadobutrol than gadoteric acid (Figure 2). The larger increase in *r*_1_ in CSF for gadobutrol compared to gadoteric acid could possibly be explained by the increased number of free hydroxyl groups in gadobutrol (2) compared to gadoteric acid (0) [20]. Free hydroxyl groups can cause non‐covalent interaction with macromolecules such as albumin, which will increase the rotational correlation time, and thus also the relaxivity [21].

Additionally, we performed one T1 mapping sequence (i.e., no repeated measurements) on a second scanner (B) and observed a systematic difference in T1 times between the two scanners (Figure 3). Correspondingly, the estimated *r*_1_ also differed. Importantly, actual *r*_1_ is not different between different scanners provided that the medium, temperature, and field strength are constant, and therefore the variation rather reflects T1 measurement bias. Accordingly, we could apply a scaling factor (κ) based on the T1 time of baseline CSF, correcting for the difference in estimated *r*_1_ between the scanners, indicating that nearly all the difference can be explained by the proportional shift in 1/T1 measurements. This conclusion is further supported by the high correlation (*R* ^2^ = 0.99–1) and a slope of around 1.18 between the 1/T1 values across Gd‐concentrations between the two scanners (Figure 3), confirming the proportional nature of the discrepancy. Similarly, for gadoteric acid in CSF and the isotonic dataset, the adjusted *r*_1_ was nearly identical to the estimated *r*_1_ on Scanner B.

These findings suggest that our estimated *r*_1_ could be transferred to another scanner (C) by measuring the T1 time of baseline CSF on that system. The relaxivity to be used can then be adjusted using the scaling relation:

$$r_{1}^{C}=\frac{T_{10}^{A}}{T_{10}^{C}}r_{1}^{A}$$

This approach provides a practical method to harmonize relaxivity estimates across different MR systems. However, further investigations are needed to investigate the generalizability of this method in a larger study involving multiple MR scanners, parameter settings and field strengths.

No previous study has measured the T1 time of CSF in vitro. Instead, prior work has used water as a surrogate [8]. For T1 measurements of CSF in vitro it is however well established that the T1 time varies across MR systems and T1 mapping methods [22, 23, 24]. Our data, acquired using the VFA technique on two different 3T MRI systems, clearly demonstrates this variability for both an isotonic solution and CSF, with a similar relative inter‐scanner difference compared to previous work using the VFA technique [25]. Importantly, our measured T1 values (Table 3) fall within the range reported in earlier studies [8, 22, 23, 24], supporting the validity of our approach. Moreover, our results suggest that the T1 time of CSF may be slightly lower than that of water (Table 3). While the VFA technique is known to give a longer T1 time compared to inversion recovery (IR) methods [26] and to exhibit greater inter‐vendor variability [25], it remains a practical choice for measuring a wide range of T1 values, particularly in preparation for clinical settings where IR methods are often too time‐consuming to be practical [27, 28].

Previous studies calculating *r*_1_ of water in physiological temperature at 3T reported relaxivities of 3.2 ± 0.3 and 3.3 ± 0.2 L mmol^−1^ s^−1^ for gadobutrol [7, 8], and 2.8 ± 0.2 L mmol^−1^ s^−1^ for gadoteric acid [7]. For Scanner A, both *r*_1_ were lower, likely due to the generally higher T1 times measured by this scanner. For Scanner B, *r*_1_ were expected to be near the previous studies, given similar baseline T1 times [8]. This was true for gadoteric acid, but gadobutrol had slightly lower *r*_1_. Possible explanations for these differences include differences in solvent composition: our measurements were done in an isotonic solution, whereas the previous studies used distilled water. The difference in ion content could possibly alter the relaxivity. Nevertheless, we chose to do our experiments with an isotonic solution to more closely resemble the properties of CSF: additionally, both previous studies used the IR method for T1 mapping, whereas we used the VFA method. All together the variability between methods and systems on baseline T1 data, and subsequently the *r*_1_, motivates the application of an adjustment method that can suppress the effect.

In the setting of research on the flow of CSF and the glymphatic system in humans, where T1 mapping is a suitable method [1], gadobutrol is the most commonly used GBCA for intrathecal injection to date [1, 17, 29]. In the paper by Watts et al. [1], *r*_1_ was assumed to be 5.1 L mmol^−1^ s^−1^, which would be similar to values previously reported for plasma [7, 9, 11]. Recalculating the results from Watts et al. [1] using *r*_1_ as found in this paper, the Gd‐concentrations would have been generally higher, which will affect an analysis of the CSF‐flow. This example illustrates the importance of accurate calculations of the Gd‐concentration when estimating effects of CSF and glymphatic flow.

Intrathecal injection of a GBCA is not approved for clinical use [30] and should therefore only be used in the setting of clinical trials. Serious adverse events that form the basis for such recommendation are associated with doses greater than 1.0 mmol [31]. Intrathecal gadobutrol has been employed in larger patient cohorts where the use of doses of 0.5 mmol and below have predominantly been associated with adverse events such as headache [32, 33], with a similar frequency as seen after intrathecal injection of the CT contrast Iodixanol [34], which is approved for intrathecal use. Given the limited availability of alternative methods to study global CSF flow and the glymphatic system, the estimation of GBCA relaxivity appears to be a rational approach for optimizing currently available methods.

A limitation with this study was that the phantom experiments were not performed during constant temperature. Our temperature drop during a scan was however very small, with variations observed between repeated scans being within the errors of previous studies [11]. The scaling factor between scanners as presented above did not have as good a fit between the 2‐flip angle images as it did for the 5‐flip angle images. Previous research shows a good fit between T1 maps reconstructed from 2 or 5 flip angles, respectively [26], which was true for our study for Scanner B but not for Scanner A. A potential cause for this could be the inability to set a fixed TR on Scanner A. Furthermore, previous literature indicates that for VFA, inaccuracies can arise when using a simple spoiled gradient echo signal equation, as it is too simplified to fully explain the data [35, 36]. This can manifest as systematic differences in *r*_1_ between configurations. However, we also observe that with the same equipment and configuration, we could consistently reproduce our relaxivity estimates. A further limitation with the proposed correction method is that it depends on low inter‐individual variability in baseline CSF T1 time. However, a recent study focusing on CSF relaxation rates reported remarkably low variation between individuals [37]. The CSF used in this study was acquired at the lumbar level of patients investigated for iNPH. This might be a concern when translating our results to intracranial CSF and the general population. We assume that our samples are representative of the healthy elderly population in the context of this study, as the albumin levels were in the normal range. Furthermore, albumin levels are known to not differ between iNPH and healthy elderly controls [38]. Albumin levels are however known to be higher in lumbar CSF compared to ventricular CSF [38], where an increase in albumin causes a decrease in T1 time [37]. However, the T1 time also differs slightly in different areas of intracranial CSF, where T1 seems to be lower in the cerebral subarachnoid space compared to the ventricles [37], potentially caused by differences in protein content [37]. Our results might therefore be more generalizable to cerebral subarachnoid space CSF than ventricular CSF. The second correction step of the B1 map as performed in this study is not standard practice and cannot be applied in an in vivo situation. The main effect of this correction was that the standard error for each of the four relaxivity estimates was reduced by 0%–3% for Scanner A and 1%–3% for Scanner B. We suspect that the need for this step was exaggerated by the properties of the phantom, where the plastic containers and support for the vials introduce areas with low signal and distortion, and that for in vivo measurements this step might not be necessary.

In conclusion, we estimated the T1 relaxivity in CSF for the two GBCAs gadobutrol and gadoteric acid at 3T. The relaxivity was higher in CSF compared to the isotonic solution, especially relevant for gadobutrol. Furthermore, there was a difference in the estimated relaxivity between MR systems. To correct for this difference, there is a potential that a scaling factor derived from baseline CSF can be used. In future studies on CSF‐flow using T1 mapping after contrast injection, we recommend using a relaxivity constant for CSF rather than one estimated from water or plasma when calculating Gd concentrations.

## Funding

This work was supported by Vetenskapsrådet (2021‐0071) and Stiftelsen för Strategisk Forskning (RMX18‐0152).

## Conflicts of Interest

The authors declare no conflicts of interest.

## Data Availability

Images (both unprocessed and processed), ROIs, as well as the code used in this study are available at https://github.com/SofiaBehndig/CSF_relaxivity.
